# Buffalo Academy of Medicine

**Published:** 1901-09

**Authors:** George F. Cott

**Affiliations:** Secretary


### SOCIETY PROCEEDINGS.

### Buffalo Academy of Medicine.

*Section on Ophthalmology, Otology, Rhinology and Laryngology, June 17, 1901.*

Reported by GEORGE F. COTT, M. D., Secretary.

AT THE last session of the Nose, Throat and Ear Section of the Academy of Medicine, Dr. E. E. Blaauw exhibited a case of tuberculosis of the eye, with the following remarks:

I present to you tonight an apparently healthy young girl, 19 years old, who enjoyed good health up to December 20, 1900. Sometime previous she complained of pain and redness of the left eye. A wash and an ice bandage relieved her. She noticed flickering lights about December 21, for the first time. This sensation continued with different intensity up to the present day. Since December 20, she can see nothing with that eye. A cataract had formed within forty-eight hours without apparent cause. A trauma can be excluded. The first time I saw her was on January 17, I found the pupil without reaction, a complete cataract, slight ciliary injection and somewhat lowered tension. The perception and projection was normal. The right eye was in all respects normal. Atropin enlarged easily the pupil. Even under enlargement no change in iris tissue could be made out.

At the same time I treated a lady, aged 42, who, being healthy, had had an inflammation of her right eye, thirteen and six years before, which did not last as long as this time. Examining her for the first time February 5, when her eye had been inflamed for at least six weeks, I found a central, irregular infiltration in the parenchyma of the cornea, and another opacity coming from the temporal side—the "sclerosirende keratitis;" tension was somewhat lower. Two weeks later a new infiltration in the upper part of the cornea had appeared with many precipitations on the membrana descemetii, which resolved within some six weeks still leaving a deep ciliary injection.

I claim that both cases belong to benign forms of tuberculosis of the uveal tract. Prof. Michel was the first to teach that half of the cases of spontaneous affection of the uveal tract are of tuberculous nature. They are primary tuberculosis of the eye, in so far as no other signs of tuberculosis in the body are to be found. Lues, rheumatism and gout are excluded here.

